# Integration of infectious diseases in climate governance in the Horn of Africa: A document review of climate strategies, policies, and action plans across four countries

**DOI:** 10.1016/j.joclim.2026.100688

**Published:** 2026-05-15

**Authors:** Tessa Rose Cornell, Yusuf Abdi Hared, Clovice Kankya, Jane N. Nyambura, Mulugeta Tamire, Louise A. Kelly-Hope

**Affiliations:** a Institute of Infection, Veterinary and Ecological Sciences (IVES), University of Liverpool, Liverpool, UK; b School of Health and Welfare, Dalarna University, Falun, Sweden; c Amoud University College of Health Sciences, Borama, Somaliland; d Department of Biosecurity, Ecosystems and Veterinary Public Health (BEP), College of Veterinary Medicine, Animal Resources and Biosecurity (COVAB), Makerere University, Uganda; e Department of Community Health, School of Public Health, Amref International University, Nairobi, Kenya; f Department of Preventive Medicine, School of Public Health, Addis Ababa University, Addis Ababa, Ethiopia

**Keywords:** Horn of Africa, Climate governance, Health, Infectious diseases, Document review

## Abstract

**Introduction:**

A range of climate-sensitive diseases are endemic to the Horn of Africa, which is increasingly vulnerable to climate extremes. Global health agencies recognise the need to abandon siloed approaches to climate change and health by integrating health in climate policies, strategies, and guidelines. This structured document review aimed to examine the extent to which health, and specifically infectious diseases, are integrated into climate governance documents in this region, with a focus on Ethiopia, Kenya, Somalia, and Uganda.

**Methods:**

A desk-based search strategy identified climate governance documents from (1) open-access climate policy databases; (2) targeted websites; (3) citation chaining; and (4) a structured literature search. Data were extracted to summarise document attributes, aims, geographic scope, integration of health topics, and infectious diseases integration, rationale for inclusion, and recommendations.

**Results:**

Ninety-eight documents were included, from Ethiopia (18.4%), Kenya (63.3%), Somalia (9.2%), Uganda (6.1%), and the broader region (3.0%). Most documents were affiliated with the Government (94.9%) or intergovernmental organisations (5.1%). Dedicated human health and infectious disease sections were identified in 59.2% and 5.1% of documents, respectively. Integrated health topics included infectious diseases (89.7%; predominately malaria, cholera or acute watery diarrhoea, and typhoid fever), nutrition (55.2%), and maternal or child health (39.7%).

**Conclusions:**

Characterisation of climate governance documents in the Horn of Africa highlighted variable integration of health and infectious diseases. These results support calls for improved coherence between climate and health governance processes and evidence translation at research-policy interfaces. The structured search and review methodology may be adopted in other climate-vulnerable contexts, in collaboration with climate and health stakeholders.

## 1 Introduction

### 1.1 Health and infectious diseases integration in climate governance

The importance of integrating health into climate strategies, policies, and action plans has been emphasised by the World Health Organization (WHO) due to increasing recognition of the health impacts of climate change, the health co-benefits of climate mitigation and adaptation strategies, and the need for climate-resilient systems [1,2]. The 2024 COP29 Special Report on Climate Change and Health argues for the global health community to abandon siloed approaches to climate change and health, and for governments and policy makers to place health at the centre of sustainable climate solutions [3]. Ninety-one percent of global Nationally Determined Contributions (NDCs) to the Paris Agreement now include health considerations [4], with quality criteria established in 2024 for integrating health into NDCs [5].

Climate change and climate-driven ecosystem changes are key drivers of the emergence, transmission, and spread of climate-sensitive infectious diseases, including zoonotic, vector-borne, and soil and water-borne infections [[6], [7], [8]]. Thus, integration of infectious disease surveillance, response, and control strategies in climate adaptation and mitigation plans is warranted, to inform coordinated global efforts and knowledge exchange between climate and health stakeholders across government, public health agencies, and academia [9].

### 1.2 Climate extremes and infectious diseases in the Horn of Africa

This document review aimed to examine the extent of integration of health and infectious diseases in regional, national, and sub-national climate governance documents in the Horn of Africa. The region is particularly vulnerable to the impacts of climate extremes, including increasing temperatures and variability in rainfall patterns, and is endemic for a wide range of climate-sensitive diseases with extreme climate events leading to increased incidence of vector-borne and waterborne diseases [[10], [11], [12], [13]]. Whilst researchers have explored regional climate-related health impacts and climate adaptation strategies at community and health system levels [14], evidence of formal research-policy translation mechanisms is limited.

This review purposively focussed on Ethiopia, Kenya, Somalia, and Uganda, which are the focus of the Climate Sensitive Disease Forecasting Tool (CLIMSEDIS) project [15], and where collaborations have been previously established with University of Liverpool researchers. These countries demonstrate variable infectious disease endemicities and geoclimatic characteristics [16,17]; thus this review aimed to provide insights into potential country variations in climate governance document availability, priorities, and integration of health.

### 1.3 Rationale and objectives

To the authors’ knowledge, no review exists to date of the integration of health and infectious diseases in climate governance documents in the Horn of Africa. The Horn of Africa provides an important and underrepresented context for this work in relation to the region’s climate vulnerabilities and infectious diseases landscape.

Our primary objective was to conduct a structured document review of climate governance documents for the Horn of Africa, with a focus on Ethiopia, Kenya, Somalia, and Uganda, to examine the integration of human health topics and infectious diseases.

This review may provide insights into coherence of climate governance documents across the region, and alignment of climate and health stakeholder priorities and coordination mechanisms. These outcomes may inform actionable steps for strengthening alignment of climate and health sector governance structures.

## 2 Materials and methods

### 2.1 Research questions

The following broad research questions were pre-determined:

(1) What current climate governance documents exist for the Horn of Africa, with a focus on Ethiopia, Kenya, Somalia, and Uganda?
(2) To what extent are health and infectious diseases integrated in identified climate documents, and which actors contribute to documents?
(3) Which infectious diseases have been integrated in identified climate documents, and what is the rationale for inclusion of specified infectious diseases?

### 2.2 Information sources and search strategies

A structured document review of climate governance documents at regional (Horn of Africa), and national and sub-national levels (Ethiopia, Kenya, Somalia, and Uganda), was conducted between May and June 2025. Climate governance documents referred to documents adopted by governing bodies which describe “…*the formal and informal rules, structures, processes and systems that define and influence action on climate change*” [18].

Documents were identified from the following sources (by the specific researchers noted): (1) online open-access climate policy databases (TRC); (2) targeted open-access websites, including those associated with government ministries, regional intergovernmental organisations, and United Nations agencies (TRC, YAH, CK, JNN, MT); (3) citation chaining, by screening reference lists of identified documents (TRC); and (4) a structured literature search and citation chaining, by screening reference lists of published literature (TRC). All identified documents affiliated with national institutions were corroborated by national researchers and experts in the fields of infectious diseases and public health (YAH, CK, JNN, MT).

Climate policy databases included the following: NewClimate Institute – Climate Policy Database [19]; London School of Economics and Political Science (LSE)/ Grantham Research Institute on Climate Change and the Environment – Climate Change Laws of the world [20]; International Energy Agency (IEA) – Policies Database [21]; Climate Policy Radar [22]; United Nations Framework Convention on Climate Change (UNFCCC) – NCD Registry [23]; and UNFCCC – Long-term strategies portal [24]. Database-specific search filters were applied to identify country-specific documents with active status, as detailed in Supplementary Material.

Targeted websites included national and local government health and climate ministries, United Nations (UN) agencies [[25], [26], [27], [28]], and non-UN multi-lateral and intergovernmental organisations (East African Community (EAC) [29], Intergovernmental Authority on Development (IGAD) [30], and African Development Bank Group [31]), as detailed in Supplementary Material. Targeted websites were searched manually for keywords related to climate, governance documents, and the geographic region and countries of interest.

The structured literature search was conducted using electronic databases PubMed and Web of Science (TRC). The search strategy used Boolean operators to combine keywords as follows: (climate) AND (governance OR report* OR policy OR policies OR strateg* OR “action plan*” OR framework* OR guideline*) AND (“Horn of Africa” OR “east* Africa” OR Ethiopia* OR Kenya* OR Somali* OR Uganda* OR Africa*). The search was restricted to titles of research articles, written in English. Reference lists of identified publications were screened manually for relevant documents.

**Fig. 1:**
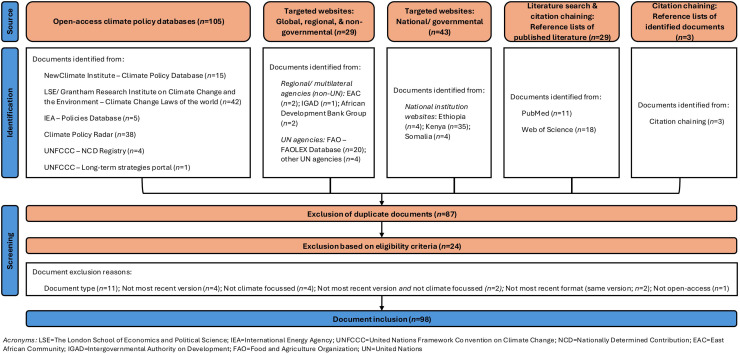
Sources, screening, and inclusion of climate governance documents in the structured document review.

Document identification, application of eligibility criteria, and final inclusion for data extraction, are documented in a flow chart (Fig. 1).

### 2.3 Selection criteria

Document inclusion criteria were as follows: (i) governance documents with a climate focus (‘climate’ specified in the title, or executive summary or equivalent section); (ii) available in English with the full text open-access; (iii) at regional (Horn of Africa or East Africa), or national or sub-national levels (Ethiopia, Kenya, Somalia, or Uganda); and (iv) published between 2010 and the search date and representing the most recent document version.

Governance document types included, but were not limited to, policies, strategies, action plans, frameworks, and guidelines, issued by national or local governments, or intergovernmental or multilateral agencies. No standard definitions for these document categories were predetermined as based on a preliminary literature review, document type definitions or rationale were not set or standardised (e.g., documents demonstrated variations by aims and objectives, context, or climate subject), and terminology used to name document types varied (e.g., strategy versus strategic plan). Open-access documents were defined as documents accessible and free of charge through web-searching.

Document exclusion criteria were as follows: (i) documents that were not climate-focused (e.g., national development plans, vision documents, or disaster risk management strategies) or were not related to governance; (ii) not available in English or not full text; (iii) involved wider geographic scope (Pan-African or global levels) or not focussing on the Horn of Africa region; and (iv) not pertaining to the year 2025. Climate project documents (specifically project proposals, progress or performance reports, or announcements), descriptive country climate profiles (affiliated with multi-lateral agencies), acts, arrangements of regulations, legal notices, and legislation documents were excluded. Based on preliminary literature review, these document types either did not relate directly to governance, or only described the provisions or deliberations of a legislative body to address a climate subject (restricting data extraction processes).

### 2.4 Data extraction and analysis

Eligible documents were exported as PDF files and searched manually for pre-defined data items and associated keywords, described in Table 1 (TRC, LAKH). Data items broadly included attributes; aims; geographic scope; integration of health or infectious diseases; rationale for infectious disease inclusion; and recommendations related to infectious disease surveillance, response, or control. Data items were extracted manually into a Microsoft Excel file for descriptive data analysis (Supplementary Material).

**Table 1:** Characteristics of climate governance documents.

Data Item	Details	Categories (if applicable)	Manual keyword search
**Document Attributes**			
**Primary affiliation**		-	-
**Contributor(s)**	Including institutions, organisations, or stakeholder groups.	-	-
**Geographic scope of primary affiliation**		Sub-national; National; Regional	-
**Type of governance document**	As reported in document title, or executive summary or equivalent section.	-	-
**Funding body(ies)**		-	-
**Publication year**		-	-
**Time period document covers**		-	-
**Document version**		-	-
**Aims or objectives**			
**Document primary aim(s)**	As reported in document executive summary or equivalent section.	-	*aim; objective*
**Document Geographic Scope**			
**Geographic scope**		Sub-national; National; Regional (Horn of Africa or Eastern Africa)	-
**Country focus**		Ethiopia; Kenya; Uganda; Somalia; Djibouti; Eritrea; Sudan; South Sudan	-
**Document Content**			
**Reference to health**	‘Health’ specified in document. a	Yes; No	*health*
**Dedicated health section(s)**	‘Health’ specified in heading(s) or sub-heading(s) of document section. a	Yes; No	
**Health topic(s)**	Health outcomes associated with climate variability and change [2,32], specified in dedicated health section(s). a	Infectious diseases; Maternal and child health; Nutrition; Mental health; Non-communicable diseases; Heat-related illness; Injury from extreme climate events; Other (specify)	-
**Dedicated infectious diseases section(s)**	‘Disease’ (or specific infectious diseases) specified in heading(s) or sub-heading(s) of document section. a	Yes; No	*infectio*
**Infectious disease(s) specified**	Infectious disease(s) specified in any part of document. a	Anthrax b; Cholera and AWD; Measles; Chikungunya; Dengue fever; Yellow fever; Rift Valley Fever b; Malaria; Typhoid fever; Leptospirosis; Viral Haemorrhagic Fevers; Hepatitis A; Fungal infections; Other (specify)	*anthra; cholera; diarrh; AWD; measles; chikungunya; dengue; yellow fever; rift; malaria; typhoid; leptospir; haemorrhagic; hemorrhagic; hepatitis; fung*
**Rationale for infectious diseases integration in dedicated health or infectious disease section(s)**		Yes; No	-
		Published literature; Priority disease selection process; Expert opinion; Other (specify)	-
**Infectious diseases recommendations in dedicated health or infectious disease section(s)**		Yes; No	-
		Surveillance; Control; Response	*surveillance; control; response; recommend*

Acronyms: WHO=World Health Organization; AWD=Acute Watery Diarrhoea.

a In reference to *human* health or disease;.

b Specify if anthrax or Rift Valley Fever described in relation to human or animal disease (additional data capture adopted following preliminary review of how these zoonotic diseases were described in documents).

Health topics were pre-determined based on established climate-related health outcomes [2]. Selected infectious diseases were pre-determined based on characterisation of climate-sensitive infectious diseases in published literature [33].

Data extractions from documents affiliated with national institutions were reviewed by respective national researchers and experts (YAH, CK, JNN, MT), to corroborate results with their country-specific knowledge.

## 3 Results

### 3.1 Document identification and inclusion

The search strategy yielded a total of 209 documents across pre-determined document sources. Following exclusion of duplicates (*n* = 87) and document exclusion following application of eligibility criteria (*n* = 24), a final number of 98 documents were included in the results. Document screening steps were summarised by Fig. 1 and Supplementary Material.

### 3.2 Document attributes, aims, and geographic scope

Ninety-eight documents related to climate governance were identified, summarised in Table 2, representing Ethiopia (*n* = 18/98, 18.4 %), Kenya (*n* = 62/98, 63.3 %), Somalia (*n* = 9/98, 9.2 %), Uganda (*n* = 6/98, 6.1 %), or multiple countries within the Horn of Africa or Eastern Africa (*n* = 3/98, 3.0 %).

**Table 2:** Climate document affiliations, types, and geographic scope in the Horn of Africa, with a focus on Ethiopia, Kenya, Somalia, and Uganda.

	National				Regional a, *N* = 3	Total, *N* = 98
Data Items	Ethiopia, *N* = 18	Kenya, *N* = 62	Somalia, *N* = 9	Uganda, *N* = 6		
**Primary affiliation category, *n* (%)**						
National Government	15 (83.3)	14 (22.6)	9 (100.0)	6 (100.0)	-	44 (44.9)
Local Government	2 (11.1)	47 (75.8)	-	-	-	49 (50.0)
Intergovernmental organisation/ Multilateral agency	1 (5.6)	1 (1.6)	-	-	3 (100.0)	5 (5.1)
**Document type, *n* (%)**						
Action Plan b	1 (5.6)	43 (69.4)	2 (22.2)	-	-	46 (46.9)
Biennial Update Report	1 (5.6)	1 (1.6)	1 (11.1)	1 (16.7)	-	4 (4.1)
Framework	-	2 (3.2)	1 (11.1)	-	1 (33.3) g	4 (4.1)
Intended Nationally Determined Contribution	1 (5.6)	1 (1.6)	1 (11.1)	1 (16.7)	-	4 (4.1)
National Adaptation Plan	1 (5.6)	1 (1.6)	-	-	-	2 (2.0)
National Communication	1 (5.6)	1 (1.6)	2 (22.2)	1 (16.7)	-	5 (5.1)
Nationally Determined Contribution	1 (5.6)	1 (1.6)	1 (11.1)	1 (16.7)	-	4 (4.1)
Policy	-	6 (9.7)	1 (11.1)	1 (16.7)	1 (33.3)	9 (9.2)
Report	3 (16.7)	1 (1.6)	-	-	-	4 (4.1)
Roadmap	2 (11.1)	-	-	-	-	2 (2.0)
Strategy c	7 (38.9)	3 (4.8)	-	1 (16.7)	1 (33.3)	12 (12.2)
Other d	-	2 (3.2)	-	-	-	2 (2.0)
**Document geographic scope, *n* (%)**						
Multi-country e	-	-	-	-	3 (100.0)	3 (3.1)
National	16 (88.9)	15 (24.2)	9 (100.0)	6 (100.0)	-	46 (46.9)
Sub-national f	2 (11.1)	47 (75.8)	-	-	-	49 (50.0)

a Regional=Horn of Africa or Eastern Africa;.

b Includes ‘Plan’ (*n* = 1) or ‘Programme of Action’ (*n* = 1);.

c Includes ‘Strategic Plan’ (*n* = 1);.

d Other: Biennial Transparency Report (Kenya *n* = 1), Guidance (Kenya *n* = 1);.

e Document relevant to multiple countries within Horn of Africa or Eastern Africa;.

f Sub-national: County-level (Kenya *n* = 47), state-level (Ethiopia *n* = 1), city administration-level (Ethiopia *n* = 1);.

g Includes ‘Master Plan’ (*n* = 1) which was described as framework in document text.

*Main affiliations* represented Government at the national (*n* = 44/98, 44.9 %) or local level (*n* = 49/98, 50.0 %), or intergovernmental organisations (*n* = 5/98, 5.1 %) comprising the East African Community (EAC; *n* = 2), the Intergovernmental Authority on Development (IGAD; *n* = 1), and the World Bank (*n* = 2).

*Document types*, as defined by individual documents, included action plans (*n* = 46/98, 46.9 %), strategies (*n* = 12/98, 12.2 %), and policies (*n* = 9/98, 9.2 %). Nationally Determined Contributions (NDCs; *n* = 4/98, 4.1 %), Intended Nationally Determined Contributions (INDCs; *n* = 4/98, 4.1 %), National Communications (NCs; *n* = 5/98, 5.1 %), Biennial Update Reports (BURs; *n* = 4/98, 4.1 %), and Biennial Transparency Reports (BTRs; *n* = 1/98, 1.0 %) represented country-level commitments to the UNFCCC to reduce national emissions and adopt climate adaptation strategies, in accordance with the Paris Agreement [34].

*The aims and objectives* of identified documents related broadly to communicating national emissions and adaptation strategies to the UNFCCC (*n* = 18/98); climate prioritisation, adaptation, and mitigation at national (*n* = 16/98) and sub-national levels (*n* = 49/98); sector-specific climate adaptation and mitigation, encompassing environment, agriculture, transport, finance, forestry, water and energy, disaster management, and education sectors (*n* = 12/98); and regional climate adaptation frameworks for EAC Partner States or IGAD Member Countries (*n* = 3/98).

*The geographic scope* of documents was predominately national (*n* = 46/98, 46.9 %) or sub-national (*n* = 49/98, 50.0 %). Documents with sub-national scope were from Kenya (county level action plans or policies, *n* = 47) or Ethiopia (regional state report, *n* = 1, or city administration action plan, *n* = 1), as shown in Table 2.

**Fig. 2:**
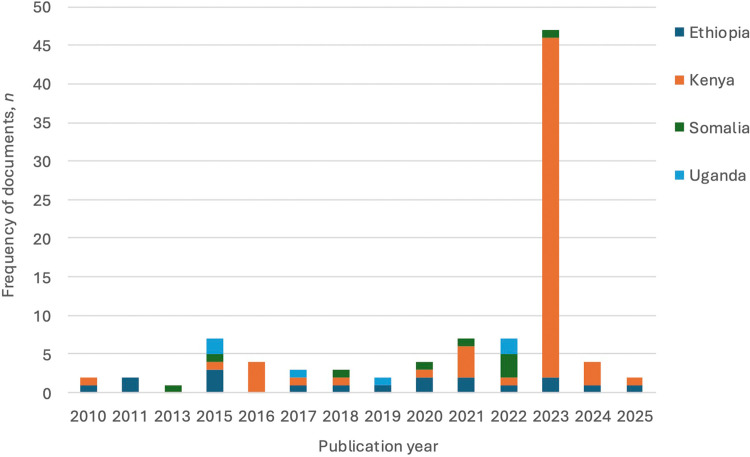
Publication year of climate documents in Ethiopia, Kenya, Somalia, and Uganda.

*Time period:* Documents were published between 2010 and 2025 and represented the most recent open-access document version (Fig. 2). The significantly higher number of documents published in 2023 represent Kenya’s County Climate Change Action Plans.

### 3.3 Integration of health and infectious diseases

The majority of documents specified health (*n* = 94/98, 95.9 %) *and* infectious disease (*n* = 78/98, 79.6 %), summarised by country in Table 3. Dedicated human health and infectious disease sections were identified in 59.2 % (*n* = 58/98) and 5.1 % (*n* = 5/98) of documents, respectively. Of the documents with dedicated health sections, 33 represented county-level climate change action plans or policies from Kenya.

**Table 3:** Integration of health or infectious diseases in climate governance documents in the Horn of Africa, with a focus on Ethiopia, Kenya, Somalia, and Uganda.

	National				Regional^a^, *N* = 3	Total, *N* = 98
Data Items, *n* ( %)	Ethiopia, *N* = 18	Kenya, *N* = 62	Somalia, *N* = 9	Uganda, *N* = 6		
**Integration of health *and* infectious diseases**^b^	8 (44.4)	56 (90.3)	7 (77.8)	4 (66.7)	3 (100.0)	78 (79.6)
**Integration of health (not infectious diseases)**^c^	9 (50.0)	4 (6.5)	1 (11.1)	2 (33.3)	0 (0.0)	16 (16.3)
**Integration of neither health nor infectious diseases**^d^	1 (5.6)	2 (3.2)	1 (11.1)	0 (0.0)	0 (0.0)	4 (4.1)

a Regional=Countries within Horn of Africa or Eastern Africa;.

b ‘Health’ and ‘disease’ specified in document, in reference to human health and human infectious diseases, respectively;.

c ‘Health’ specified in document, in reference to human health;.

d Neither ‘health’ nor ‘disease’ specified in document.

**Fig. 3:**
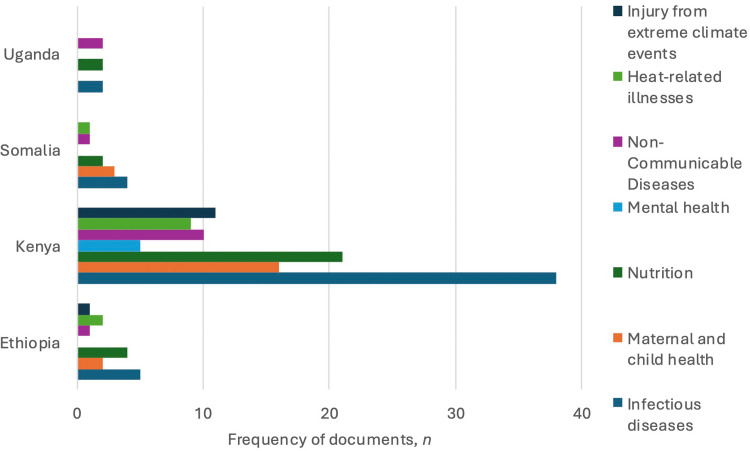
Climate-related health topics described by climate governance documents with dedicated human health sections, in Ethiopia, Kenya, Uganda, and Somalia.

Climate-related health topics described in dedicated human health sections included: infectious diseases (*n* = 52/58, 89.7 %); nutrition (*n* = 32/58, 55.2 %); maternal or child health (*n* = 23/58, 39.7 %); non-communicable diseases, comprising respiratory and nutrition-related diseases (*n* = 16/58, 27.6 %); heat-related illnesses (*n* = 15/58, 25.9 %); injury from extreme climate events (*n* = 12/58, 20.7 %); and mental health (*n* = 6/58, 10.3 %). Supporting data are available in Supplementary Material and Fig. 3.

**Fig. 4:**
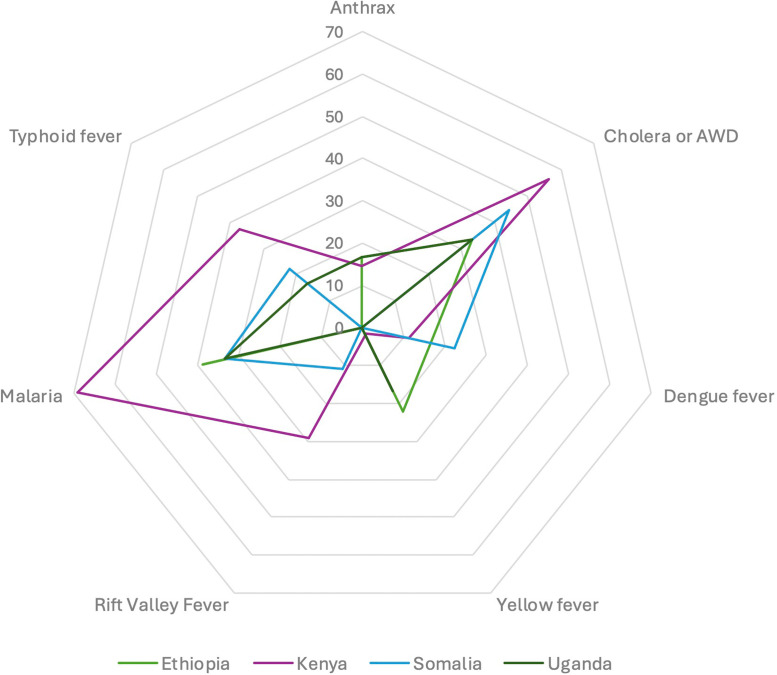
Radar chart of percentage of governance documents specifying climate-sensitive infectious diseases, in Ethiopia, Kenya, Somalia, and Uganda. Acronym: AWD=Acute Watery Diarrhoea.

Across all documents (with and without dedicated human infectious disease sections), human infectious diseases most frequently specified were: malaria (*n* = 58/98, 59.2 %); cholera or acute watery diarrhoea (AWD; *n* = 50/98, 51.0 %); typhoid fever (*n* = 28/98, 28.6 %); Rift Valley Fever (RVF; *n* = 21/98, 21.4 %); dengue fever (*n* = 14/98, 14.3 %); anthrax (*n* = 13/98, 13.3 %); and yellow fever (*n* = 8/98, 8.2 %; Fig. 4). Specification of RVF or anthrax in relation to human and animal disease were recorded. Overall, infectious disease categories were categorised as vector-borne, food-borne, zoonotic, waterborne, and climate-sensitive or climate change-related.

Within dedicated human health or infectious disease sections, rationale for the inclusion of infectious diseases were provided for 72.4 % (*n* = 42/58) of documents. Rationale comprised: descriptions of the disease landscape (at national and sub-national levels) and associations between climate and infectious diseases, without provision of references to literature (*n* = 34/42, 81.0 %); and references to published literature or data sources affiliated with national or local Government Ministries and institutions, and regional or international inter-governmental organisations (*n* = 14/42, 33.3 %; supplementary material).

### 3.4 Infectious disease recommendations

Within dedicated human health or infectious disease sections, recommendations were provided by 75.9 % (*n* = 44/58) of documents, relating to infectious disease surveillance (*n* = 28/44, 63.6 %), response (*n* = 21/44, 47.7 %), and control (*n* = 37/44, 84.1 %), with country-specific actions available in Supplementary Material. A summary of recommendations described in documents is presented in Table 4 for all four countries.

**Table 4:** Examples of recommendations for human infectious disease surveillance, response, and control, from Ethiopia, Kenya, Uganda, and Somalia.

Infectious disease strategies	Strategies described in climate governance documents
**Surveillance**	• Surveillance measures and monitoring of climate change related diseases to enhance early warning systems • Integration of health and environmental surveillance protocols to undertake disease surveillance • Assessment of population risks to climate change using climate-disease prediction models • Data sharing for climate-sensitive diseases • Community disease surveillance team capacity • Collection of community-sourced health data on vector-borne diseases • Incorporation of gender-disaggregated indicators into disease surveillance protocols
**Response**	• Health infrastructure improvements to assist in early diagnosis and treatment of climate change related diseases • Rapid responses to control epidemics • Development of health programmes, protocols, and guidance to manage new climate change related diseases
**Control**	• Climate-sensitive disease prevention program design • Promotion of climate change related disease awareness and prevention • Safe water chain and sanitation facilities to limit waterborne disease outbreaks • Vaccination programmes • Identification of vulnerable groups for climate-sensitive diseases • Vector control and prevention strategies • Malaria prevention (long lasting insecticide treatment nets [LLITNs] distribution and coverage)

## 4 Discussion

### 4.1 Main outcomes

This review indicated that integration is neither universal nor comprehensive, with 59.2 % and 5.1 % of identified documents containing dedicated health and infectious diseases sections, respectively. This highlights a potential need to develop a more standardised approach, in collaboration with national stakeholders, to address inconsistencies in how climate-sensitive health outcomes are integrated into governance documents or how effectively evidence is translated at research-policy interfaces. Whilst the impact of climate extremes on other sectors (e.g., agriculture) has been a greater focus of climate policies [35], our outcomes support calls for greater integration of health in sustainable climate governance structures [3] and improved climate and health stakeholder knowledge exchange [9]. This requires effective cross-sectoral and multidisciplinary coordination mechanisms.

Human infectious diseases most frequently specified in identified documents (including malaria, cholera or AWD, typhoid fever, and RVF) reflect regional climate-sensitive infectious disease priorities and concerns [36]. The quality of extracted data on infectious disease priorities and recommendations was not standardised between countries and document types, which may reflect the variable aims of these climate-focussed documents. Rationale for inclusion or prioritisation of infectious diseases, including prioritisation processes (e.g., whether disease priorities were informed by surveillance mechanisms, research, or formal consultation processes), were not consistently stated. These variations provide evidence to strengthen science-policy dialogue, and to promote cross-sectoral data sharing and more integrated approaches to climate change and health [1,3].

This review presents a structured approach for examining the integration of health and infectious in climate governance documents, which could be adopted and adapted by other countries and regions. The collation of key climate sources into an information database is a useful resource for current and future country comparisons, especially in the context of a changing climate [8]. The lists of document sources and types are non-exhaustive and may be developed, in consultation with stakeholders.

### 4.2 Country variations in climate governance

This review highlighted a higher number of documents for Kenya. In accordance with Kenya’s Climate Change Act, 2016 [37], County Climate Change Action Plans were developed using consultative approaches which engaged county-level stakeholders. These documents provide valuable descriptive data on the epidemiology of infectious diseases at the sub-national level and may provide a template for other countries to establish climate-related health and infectious disease priorities, and response strategies, at sub-national levels. Our outcomes add to previous evaluations of Kenya’s County-level Integrated Development Plans, of which 48.9 % mentioned health in the context of climate change [38].

Limited sub-national documents were identified for the other countries of focus and the integration of sub-national disease priorities into existing national documents should be examined. Inter-country variations may be explained by variations in the structure and robustness of climate governance structures, exemplified by examination of barriers to climate policy integration in Ethiopia [39]. Furthermore, documents without a climate focus (as defined by this review) but with embedded sections on climate (e.g., disaster management strategies or national development plans) were excluded. Development of sub-national documents would support calls for improved multilevel climate governance, including engagement of community-level actors in climate policy formulation [[40], [41], [42]].

### 4.3 Limitations

The search was limited to English-language documents. Non-English-language documents were not identified; however, the potential bias of selected literature sources towards identifying English-language documents is acknowledged, which may have resulted in exclusion of national documents in local languages.

Although this review aimed to identify documents with a climate focus, only “climate” was used as a keyword for the structured literature review leading to potential exclusion of documents which used climate synonyms or related terminology (e.g., specific extreme climate events) and documents with broader scope addressing climate-related topics.

### 4.4 Future directions

Improved cross-sectoral governance will optimise surveillance, response, and control strategies for climate-sensitive diseases. This is important for the climate-vulnerable Horn of Africa region, which is endemic for climate-sensitive diseases exacerbated by large scale climate phenomena such as the El Niño Southern Oscillation [10,43,44].

Existing tools for health integration in climate documents could be developed, in collaboration with key stakeholders at regional, national, and sub-national levels, and should consider context-specific enablers of or barriers to integration (e.g., variable governance structures, degrees of cross-sectoral collaboration, and political commitments) [45]. Furthermore, published research or data sources which underpin governance decisions on climate-sensitive health outcomes, including infectious disease priorities, should be referenced in documents. In addition, research directions may be informed by existing policies and related gaps (e.g., measuring the impact of climate governance structures on mitigating disease risk [46]).

## 5 Conclusion

Collation and characterisation of key climate governance documents across four countries in the Horn of Africa have highlighted that the integration of health and infectious diseases, and the nature and extent of integration, are not standardised. These outcomes support calls for improved cross-sectoral coordination, coherence between climate and health governance processes, and evidence translation at research-policy interfaces.

The search strategy may be applied in other contexts which are vulnerable to the impacts of climate change and climate-sensitive diseases, to produce a structured database of key climate documents. This may be developed to include additional document sources and types in consultation with national climate and health stakeholders.

## Funding

This work was supported by the Climsedis Sensitive Disease Forecasting Tool (CLIMSEDIS) research project, funded by the Wellcome Trust (grant number 225997/Z/22/Z).

## CRediT authorship contribution statement

**Tessa Rose Cornell:** Writing – review & editing, Writing – original draft, Visualization, Validation, Project administration, Methodology, Investigation, Formal analysis, Data curation, Conceptualization. **Yusuf Abdi Hared:** Writing – review & editing, Validation, Investigation. **Clovice Kankya:** Writing – review & editing, Validation, Investigation. **Jane N. Nyambura:** Writing – review & editing, Validation, Investigation. **Mulugeta Tamire:** Writing – review & editing, Validation, Investigation. **Louise A. Kelly-Hope:** Writing – review & editing, Validation, Supervision, Methodology, Investigation, Funding acquisition, Formal analysis, Conceptualization.

## Declaration of competing interest

The authors declare that they have no known competing financial interests or personal relationships that could have appeared to influence the work reported in this paper.

## Appendix Supplementary materials

Image, application 1

## Footnotes

Supplementary material associated with this article can be found, in the online version, at doi:10.1016/j.joclim.2026.100688.
